# All-Optical Artificial Synapse Based on ε-Ga_2_O_3_ and β-Ga_2_O_3_ Mixed-Phase Thin Films

**DOI:** 10.3390/ma19040711

**Published:** 2026-02-12

**Authors:** Jiale Niu, Zixuan Liu, Xuewen Ding, Zhang Meng, Xianxu Li, Jiajun Deng, Wenjie Wang, Fangchao Lu

**Affiliations:** 1 Institute of Clusters and Low Dimensional Nanomaterials, School of Mathematics and Physics, North China Electric Power University, Beijing 102206, China; jiale_n0913@163.com (J.N.); 18906360296@163.com (Z.L.); 120242209099@ncepu.edu.cn (X.D.); mengz0612@gmail.com (Z.M.); djiaj@ncepu.edu.cn (J.D.); wwj2008@ncepu.edu.cn (W.W.); 2 School of Physics and Astronomy, Beijing Normal University, Beijing 102206, China; 19862509808@163.com; 3 Hebei Key Laboratory of Physics and Energy Technology, North China Electric Power University, Baoding 071000, China

**Keywords:** gallium oxide (Ga_2_O_3_), chemical vapor deposition (CVD), ε/β-Ga_2_O_3_ mixed-phase films, all-optical artificial synapse

## Abstract

All-optical memristors possess light-sensing and storage capabilities while simultaneously simulating human synaptic functions, demonstrating immense potential in the field of brain-inspired computing for realizing bionic synapses and brain-like intelligence. In this work, we successfully produced ε-Ga_2_O_3_ films, ε/β-Ga_2_O_3_ mixed-phase films, and β-Ga_2_O_3_ films via chemical vapor deposition (CVD). The optical output and optical response characteristics of the thin films are investigated under 254 nm and 365 nm lasers. The CVD-grown ε-Ga_2_O_3_ is found to process a small amount of defects and insignificant memristive properties and the β-Ga_2_O_3_ obtained from the annealing of ε-Ga_2_O_3_ exhibits superior crystal quality but lacks memristive properties, while the ε/β-Ga_2_O_3_ mixed-phase films grown directly by CVD contain a fair amount of defects and demonstrate persistent resistance retention exceeding 104 s. Based on the excellent memristive properties of ε/β-Ga_2_O_3_ mixed-phase films, we conducted experiments simulating optical synapses. By adjusting optical pulse parameters (intensity, repetition rate, and duration), we successfully modeled the short-term plasticity (STP) and long-term plasticity (LTP) observed in biological synapses. Experiments confirm that light stimulation can effectively induce synaptic behaviors, such as the progressive conversion of short-term memory (STM) into long-term memory (LTM), and further fully reproduce the neuroplasticity process of “learning-forgetting-relearning.” This study demonstrates a photoconductive synapse memristor based on the wide-bandgap material gallium oxide, exhibiting exceptional air stability with sustained photoconductivity maintained for over a year. This study provides new insights into the practical application feasibility of all-optical artificial synapses based on gallium oxide.

## 1. Introduction

With the advent of the era of artificial intelligence and big data, the traditional von Neumann computing architecture faces increasingly prominent challenges due to the separation of storage and computation and high energy consumption [1,2,3]. Neuromorphic computing offers a revolutionary pathway to achieving energy-efficient, parallelized novel computing paradigms by simulating the dynamic plasticity of neurons and synapses [4,5,6]. Among these, artificial synapses serve as the fundamental building blocks of neural networks, with their performance directly determining the functionality and complexity of neuromorphic systems. In recent years, a wide variety of neuromorphic devices primarily relying on electrical stimulation have emerged in large numbers. Although these artificial synapses provide a promising hardware foundation for the development of neuromorphic computing chips, inherent issues such as electrical crosstalk limit their operational speed and response bandwidth [7,8,9,10,11]. Significant progress has been made in developing artificial synapses based on structures such as memristors, floating-gate transistors, and photoconductive devices. Among these, oxide semiconductors (e.g., ZnO, TiO_2_) have been extensively studied due to their ion migration and oxygen vacancy regulation mechanisms [12,13]. However, existing devices mostly rely on electrical stimulation to update synaptic weights, which leads to issues such as electrical crosstalk and high energy consumption [14]. Optical synapse devices utilize light as the stimulus source instead of electrical pulses, enabling neuromorphic systems with high-speed transmission, high bandwidth, low crosstalk, and greater energy efficiency. This represents a crucial direction for overcoming the limitations of electrical synapses, demonstrating unique advantages particularly in all-optical neuromorphic systems [8,14,15,16,17,18,19,20].

Gallium oxide (Ga_2_O_3_), as an emerging ultra-wide bandgap semiconductor (bandgap width approximately 4.8–5.3 eV), possesses high breakdown electric field, excellent photoelectric response, and good chemical stability. It has garnered significant attention in the fields of day-blind ultraviolet photodetection and power devices. Its rich crystal phase structures (such as β, ε, α, γ, etc.) provide ample opportunities for band structure engineering and regulation of optoelectronic properties. Among these, the ε phase (orthorhombic crystal system) exhibits spontaneous polarization and ferroelectric properties due to its non-centrosymmetric structure [21], while the β phase (monoclinic crystal system) possesses outstanding structural stability [22]. Research on gallium oxide in the field of neuromorphic devices remains in its early exploratory stages. The current mainstream strategy involves constructing heterojunctions with other materials to simulate artificial synapse functionality [23,24,25]. Compared to directly utilizing epitaxial gallium oxide single crystals for fabricating optoelectronic devices, the introduction of heterojunction structures can indeed expand device functionality and performance. However, their fabrication process involves critical steps such as hetero-material integration, interface engineering, and stability control, significantly increasing process complexity and technical difficulty. To date, research on gallium oxide materials grown by direct epitaxy has primarily focused on the electrically stimulated memristive behavior of amorphous or β- Ga_2_O_3_ [21,26]. However, systematic exploration of the memristive properties of ε-phase gallium oxide grown by direct epitaxy remains lacking, and relevant research is still relatively scarce [21]. Gallium oxide thin films containing mixed β and ε crystalline phases, prepared via methods such as CVD, may significantly influence the separation, transport, and capture kinetics of photogenerated carriers through band bending at phase boundaries, defect state distribution, and modulation of the built-in electric field. It is anticipated that this approach will provide a novel physical mechanism for simulating complex synaptic plasticity, thereby offering an ideal material platform for constructing high-performance optical synapse devices. How to precisely control ε/β mixed phases through controlled CVD growth and, based on this, develop fully optically operated neuromorphic devices to simulate bionic cognitive processes—including learning, forgetting, and relearning—represents a highly cutting-edge and challenging research topic.

This study successfully fabricated gallium oxide thin films with ε/β mixed crystal phases via CVD to address the aforementioned challenges, and based on this, constructed an all-optical neuromorphic synapse device. By leveraging interface effects and defect engineering within a hybrid-phase structure, this device achieves synaptic weight modulation entirely through optical pulses without requiring electrical signals. It can simulate the plasticity behaviors of biological synapses, such as STP and LTP. All-optical operation not only effectively avoids electrical crosstalk and reduces static power consumption, but also fully leverages the advantages of optical signals—high bandwidth and low latency—providing a viable path for constructing energy-efficient, high-speed brain-inspired computing systems. Notably, the device exhibits persistent resistance retention exceeding 104 s, with this characteristic maintained for over a year, demonstrating exceptional non-volatility. This provides crucial support for its application in long-term learning and memory storage. Furthermore, this study successfully simulated the “learning-forgetting-relearning” dynamic process of biological synapses in gallium oxide devices with multiple crystalline phases, demonstrating key features such as the transition from STM to LTM, the natural decay of memory, and the rapid reconstruction of memory through re-stimulation. This work not only demonstrates the application potential of ε/β mixed-phase gallium oxide in all-optical neuromorphic devices but also provides novel insights into material design and device construction for developing next-generation optoelectronic synapses capable of simulating higher-level cognitive functions.

## 2. Materials and Methods

This study employs CVD technology to grow gallium oxide films on single-sided polished sapphire (0001) substrates. Using 99.99% pure gallium oxide powder supplied by McLean (Shanghai, China) as the precursor material, three samples—ε-Ga_2_O_3_, ε/β-Ga_2_O_3_, and β-Ga_2_O_3_—were prepared and designated as E, EB, and B, respectively. The preparation process for samples E and EB is as follows: Place the substrates approximately 1 cm and 2 cm from the right-hand insulation layer, respectively. Position the ceramic boat coated with gallium oxide powder approximately 7 cm upstream from the substrates. Using an Ar gas mixture containing 10% H_2_ as the carrier gas, raise the temperature uniformly to 1000 °C over 40 min (heating rate approximately 25 °C/min) and maintain this temperature for 40 min. During this stage, the gas flow rate was 200 sccm. Subsequently, gas supply was stopped, and the sample was removed after natural cooling to room temperature with the furnace. Preparation process for Sample B: The prepared Sample E film was annealed at 1000 °C under vacuum for 40 min to obtain Sample B with improved surface flatness [26].

X-ray diffraction (XRD) analysis was performed on these samples using a SmartLab SE instrument (Rigaku, Japan) to determine their structural characteristics. Raman spectroscopy analysis was conducted using an iHR550 spectrometer (HORIBA Instruments Incorporated, Salt Lake City, UT, USA) to characterize the physical phases. All electrical and optoelectronic properties were measured using a probe station equipped with an ultraviolet light source. Lasers at 254 nm and 365 nm were employed as light sources for optoelectronic measurements.

## 3. Results

**Figure 1:**
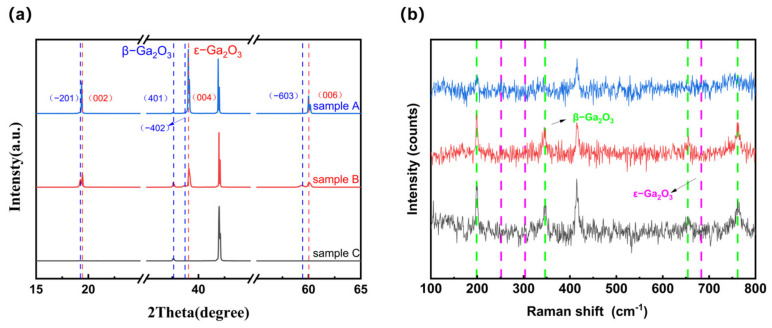
(**a**) XRD patterns of samples E, EB, and B; (**b**) Raman spectra of samples E, EB, and B.

Figure 1a shows the XRD patterns of samples E, EB, and B prepared in this experiment. Both samples E and B exhibit ε-Ga_2_O_3_ peaks at 19.35°, 39.03°, and 60.00°, corresponding to the (002), (004), and (006) crystal planes of ε-Ga_2_O_3_, respectively. Additionally, sample EB exhibits peaks characteristic of β-Ga_2_O_3_ at 19.24°, 37.59°, 38.67°, and 59.33°, corresponding to the (−201), (401), (−402), and (−603) crystal planes of β-Ga_2_O_3_. XRD provides relatively accurate content analysis. Based on the peak area calculation, it can be inferred that the composition ratio of β-Ga_2_O_3_ to ε-Ga_2_O_3_ in sample EB is approximately 1:1. Sample B exhibits a peak characteristic of β-Ga_2_O_3_, showing a weaker peak on the (−201) crystal plane but a stronger peak on the (401) crystal plane. This peak is characteristic of β-Ga_2_O_3_ formed by the transformation of ε-Ga_2_O_3_ from sample E during annealing [27,28]. Figure 1b shows the Raman spectra corresponding to these three samples, where the green dashed line corresponds to the peak of β-Ga_2_O_3_, and the pink dashed line corresponds to the peak of ε-Ga_2_O_3_. Among these, samples EB and B exhibited distinct peaks associated with the Ag mode of β-Ga_2_O_3_ at 199.936, 347.723, 654.046, and 761.612 cm^−1^. Weak peaks associated with the ε-Ga_2_O_3_ A1 mode were observed in samples E and EB at 248.9304 and 679.3 cm^−1^. It can thus be seen that both β-Ga_2_O_3_ and ε-Ga_2_O_3_ coexist in sample EB [28].

Regarding the surface roughness and film thickness of Samples E, EB, and B, Figure S1a,b present the AFM and SEM images for the three samples, respectively. Analysis of Figure S1b yields Rq values of 2.984 nm, 0.109 nm, and 0.057 nm for samples E, EB, and B, respectively. Figure S1c shows the film thicknesses of Sample E, Sample EB, and Sample B to be 823 nm, 903 nm, and 992 nm, respectively.

**Figure 2:**
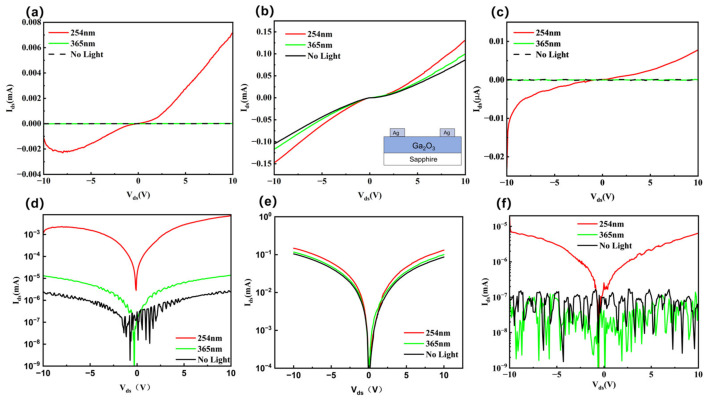
I-V characteristics of Ag/Ag electrodes on gallium oxide films. Linear relationship: (**a**) Sample E, (**b**) Sample EB, (**c**) Sample B, where the inset in (**b**) shows a schematic of the sample structure. Logarithmic relationship: (**d**) Sample E, (**e**) Sample EB, (**f**) Sample B.

Figure 2 shows the ultraviolet response characteristics of samples E, EB, and B. As shown in the inset of Figure 2b, an Ag electrode was deposited onto the Ga_2_O_3_ film to fabricate a metal–semiconductor–metal (MSM) structure (see Figure S1c for detailed sample dimensions). Two types of ultraviolet light sources were employed in the experiment, with wavelengths of 254 nm and 365 nm, respectively. Figure 2 shows the current–voltage characteristics of samples E, EB, and B under three different illumination conditions: 254 nm, 365 nm, and no illumination, using both linear and logarithmic coordinate systems. As shown in Figure 2a,d, under 365 nm illumination, the photocurrent of sample E increased by one order of magnitude compared to the dark current, while under 254 nm illumination, the photocurrent-to-dark-current ratio increased by three orders of magnitude. In contrast, the MSM structure of sample EB shown in Figure 2b,e exhibits current increases with rising voltage under both dark conditions and 365 nm illumination. Furthermore, under 254 nm illumination, the photocurrent shows only a modest enhancement compared to the dark current. Furthermore, under 254 nm illumination, the photocurrent shows only a modest increase compared to the dark current. Sample B exhibits nearly identical photocurrent and dark current under 365 nm illumination, indicating a non-conductive state. However, under 254 nm illumination, the photocurrent increases significantly, as shown in Figure 2c,f. We know that the increase in conductivity under light irradiation below the bandgap energy is due to photoelectrons emitted from defects. Therefore, Sample E and Sample B exhibit low defect concentrations, whereas Sample EB has a comparatively higher defect concentration. On the other hand, under 254 nm illumination, the photocurrent of samples E, EB, and B all increased to a certain extent, indicating that interband carrier transitions play a primary role.

**Figure 3:**
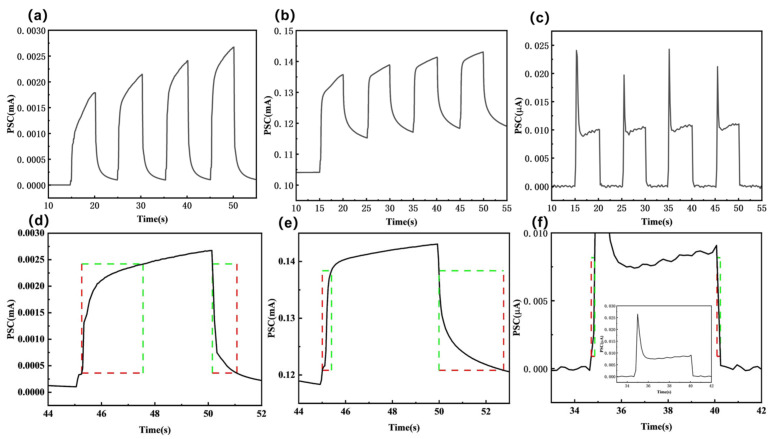
Photoresponse to 254 nm ultraviolet light at 10 V: (**a**) Sample E, (**b**) Sample EB, (**c**) Sample B; Light-induced experimental curves of current rise and decay: (**d**) Sample E, (**e**) Sample EB, (**f**) Sample B.

Figure 3a–c show the multiple-stimulus current response curves of samples E, EB, and B under 10 V voltage and 254 nm illumination. After multiple light-cycle tests, the photocurrent peak of sample E exhibited a regular increase, and upon cessation of illumination, the photocurrent rapidly declined to a level close to its initial state. In contrast, while sample EB also exhibited a regular increase in photocurrent peak, its recovery behavior after illumination cessation differed significantly. The photocurrent did not immediately return to baseline but first rapidly declined to a transient plateau before entering a slow decay phase. The overall time required for the photocurrent to recover to its initial level was markedly longer, demonstrating persistent resistance retention exceeding 104 s. This difference indicates that both samples E and EB contain a large number of defect states. However, the defect states in sample EB exhibit not only a higher density and deeper energy levels but also a more pronounced persistent photocurrent effect. This property endows it with significant potential for simulating biological synapses and memory behaviors, with this characteristic persisting for over a year, as demonstrated in Figure S2a–c (Supplementary Information). It exhibits outstanding nonvolatility, leading us to conclude that sample EB is an excellent candidate material for constructing memristor devices. As shown in Figure 3c, under illumination, sample B exhibits an initial current overshoot phenomenon, characterized by the photocurrent rapidly reaching its peak before stabilizing.

To further investigate the performance of photodetectors for samples E, EB, and B, their transient response to 254 nm illumination was measured at 10 V. The response (rise) and recovery (decay) obtained within a single switching cycle are shown in Figure 3d–f. Calculations yielded the following values for sample E: rise time (time taken for current to increase from 10% to 90% of maximum photocurrent), response rate, and fall time were 2.30 s, 3.85 mA/W, and 0.92 s, respectively. For sample EB, the rise time, response rate, and fall time were 0.39 s, 12.63 mA/W, and 2.77 s, respectively. For sample B, the rise time, response rate, and fall time were 0.15 s, 0.012 mA/W, and 0.14 s, respectively. The rise time and decay time are primarily influenced by two factors: first, the contribution of interband optical transitions to carrier concentration during switching states; second, the trapping and release processes of relevant carriers by defects within the sample [29]. Additionally, several studies have demonstrated that improving optical response time can also be achieved through annealing treatment [30], the use of phototransistors [31], and argon plasma gas treatment [32].

**Figure 4:**
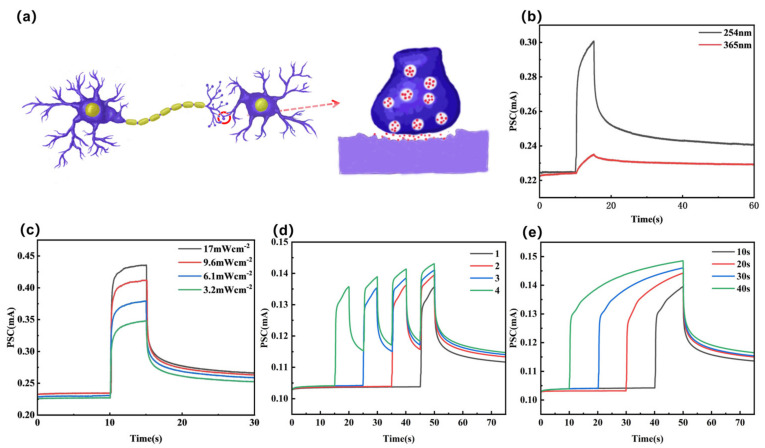
(**a**) Schematic diagram of a biological neural synapse; Current-time characteristics of the MSM device under different (**b**) wavelengths, (**c**) pulse intensities, (**d**) pulse numbers, and (**e**) pulse durations of light irradiation.

This device exhibits wavelength-dependent response variations in the deep ultraviolet range that resemble those of biological neural synapse systems. As the fundamental structural unit connecting neurons in the human brain, biological synapses play a crucial role in transmitting chemical signals and triggering nerve impulses. A schematic diagram of their biological structure is shown in Figure 4a. Biological synapses play a crucial role in regulating neural circuit signaling by connecting presynaptic and postsynaptic neurons. When stimulated, the presynaptic membrane releases neurotransmitters that bind to receptors on the postsynaptic neuron, thereby triggering a postsynaptic current (PSC) [25,33,34]. PSC is determined by synaptic weights and is crucial for the transmission, processing, and storage of information within the nervous system. This mechanism is central to the fundamental operation of synaptic activity and forms the basis of neural communication [25,35,36]. The MSM structure depicted in the inset of Figure 2b consists of a silver electrode, sample EB, and a silver electrode, corresponding to the presynaptic membrane, synaptic cleft, and postsynaptic membrane of the synapse, respectively.

As shown in Figure 4b, we observed that the photocurrent response of the sample under 254 nm light excitation was higher than that under 365 nm excitation. Furthermore, both responses exhibited nonvolatile residual behavior after the light stimulus ceased (i.e., a small current persisted even after the voltage returned to zero). In oxide semiconductors, the slow decay of photocurrent typically originates from strong photoelectric coupling effects, whose primary mechanism is closely related to the slow recombination process of photoelectrons generated by the ionization of neutral oxygen vacancies (VOS) [37,38,39]. Although the bandgap of the materials used in this study exceeds the corresponding photon energy, hole-mediated transitions can still achieve ionization of neutral VOS [39,40]. Under this mechanism, persistent photocurrent effects can still occur even at lower photon energies, providing a reasonable explanation for the device’s response behavior to both deep ultraviolet light sources [39].

The oxygen vacancies in the sample EB continuously capture and release charge carriers, thereby effectively modulating the current. Consequently, the resulting MSM device also exhibits outstanding optical analog synapse-like neuromorphic functionality. During the process of light turning on and off, this device effectively mimics the learning and forgetting behavior characteristic of biological synapses. We conducted a systematic investigation of synaptic plasticity in the device by applying 254 nm light pulses of varying intensities, frequencies, and widths. Figure 4c shows the photocurrent response measured when applying single light pulses of varying intensities. In this context, light turning on corresponds to the learning process, while light turning off corresponds to the forgetting process. When the light is turned on, a photocurrent exhibiting exponential growth can be observed; when the light source is turned off, the photocurrent gradually decays. We observed that the photocurrent intensified with increasing pulse intensity, a phenomenon similar to biological synapses. We believe this device integrates sensing and memory functions, enabling the acquisition and storage of optical information. To further investigate this phenomenon, we varied the number of optical pulses applied to the device during synaptic learning, administering 1, 2, 3, and 4 pulses respectively. The experimental results are shown in Figure 4d. By increasing the number of light pulses, *ΔI*_r_ was observed to rise from 0.135 mA after a single pulse to 0.143 mA after four pulses. When illumination was turned off for 25 s, the current increased from 0.112 mA to 0.115 mA, indicating a transition from STM to LTM. Modifying the duration of light pulses applied to synapses undergoing learning similarly facilitates the transition from STM to LTM. The corresponding results are shown in Figure 4e, exhibiting a trend similar to that observed with the number of light applications. When the illumination duration increased from 10 s to 40 s, the *ΔI_r_* of the device rose from 0.139 mA to 0.149 mA. Thirty-five seconds after illumination ceased, the current increased from 0.114 mA to 0.117 mA.

**Figure 5:**
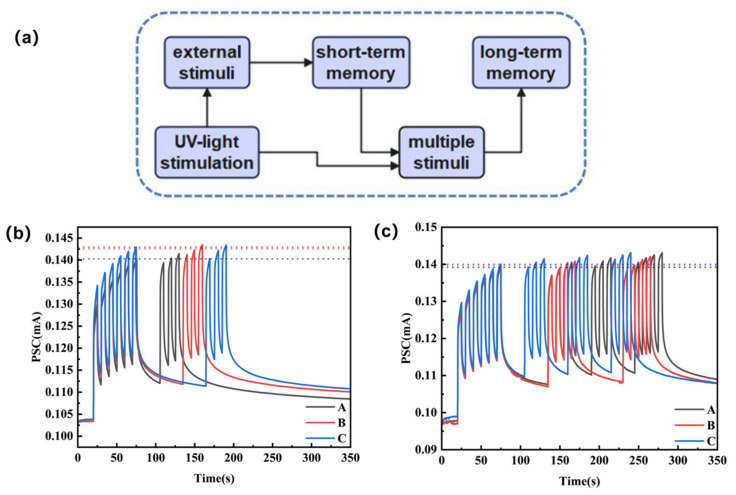
(**a**) Schematic diagram of learning–forgetting–relearning behavior; (**b**) Relearning processes at different forgetting intervals after 6 cycles of learning, where A denotes 6 learning sessions, a 30-s pause, followed by 3 relearning sessions; B denotes 6 learning sessions, a 1-min pause, followed by 3 relearning sessions; C denotes 6 learning sessions, a 1-min-30-s pause, followed by 3 relearning sessions; (**c**) Different “forgetting-relearning” patterns after 6 cycles of learning: A denotes 6 learning sessions, 1-min forgetting, followed by 3 relearning rounds with 30-s intervals; B denotes 6 learning sessions, 1-min forgetting, followed by 2 relearning rounds with 1-min intervals; C denotes 6 learning sessions, 30-s forgetting, followed by 3 relearning rounds with 30-s intervals; (The horizontal lines in Figure 5a,b represent the PSC achieved after the initial 6 learning sessions).

The “learning-forgetting-relearning” behavior represents a comprehensive memory storage model [41], primarily encompassing two major memory types in psychology: STM and LTM. As shown in Figure 5a, this device simulates the memory formation process in biological neural systems: external stimulus information is first rapidly stored as STM, based on transient and weak reinforcement of synaptic connections that typically lasts only seconds to minutes. Subsequently, during natural decay, repeated learning occurs under periodic optical pulse stimulation. Through repeated training and reinforcement, STM can gradually strengthen and transform into stable LTM, transferring to the cerebral cortex for enduring storage. Due to the device’s exceptional optical synapse properties, it successfully reproduces the complex memory behavior encompassing the dynamic process of “learning-forgetting-relearning” under continuous light pulse modulation [41]. We experimented with devices undergoing different durations of the forgetting process under identical learning conditions, then measured the magnitude of the photocurrent after three subsequent relearning cycles to calculate the change in photocurrent. As shown in Figure 5b, applying six continuous optical pulses as the learning process resulted in a significant increase in the optical response current with the number of pulses. Subsequently, learning was repeated after periods of light extinction lasting 30 s, 60 s, and 90 s. As the forgetting period lengthened, the calculated photocurrents achieved after three additional learning cycles increased by 1.26 μA, 0.95 μA, and 0.54 μA, respectively, compared to the initial six-cycle learning. The photocurrents reached 0.1414 mA, 0.1435 mA, and 0.1434 mA, respectively. Therefore, it can be concluded that, under the same initial learning conditions, the longer the forgetting interval, the weaker the level of memory retention achieved after relearning. We observed variations in the magnitude of photocurrent achieved after six light stimulations across three experiments, attributable to differing contact positions between the measurement device and electrodes. Building upon this foundation, we further investigated the process of repeated “forgetting-relearning” under various conditions. As shown in Figure 5c, under identical initial learning conditions, shorter forgetting intervals and greater learning intensity (learning duration and frequency) result in stronger memory retention and slower forgetting rates. This series of experiments demonstrates that long-term learning enhances memory strength, while increasing the number of learning sessions accelerates learning speed and slows down the rate of forgetting [42,43]. It can thus be observed that by repeating the learning–forgetting–relearning cycle and rationally designing the period or pulse frequency for each learning phase, synaptic weights can be stimulated to achieve the learning objective—that is, the desired memory level expressed as a specific current value. The device’s light-induced memory and forgetting properties closely resemble those of visual synapses, laying the foundation for the realization of artificial synaptic systems.

## 4. Discussion

**Figure 6:**
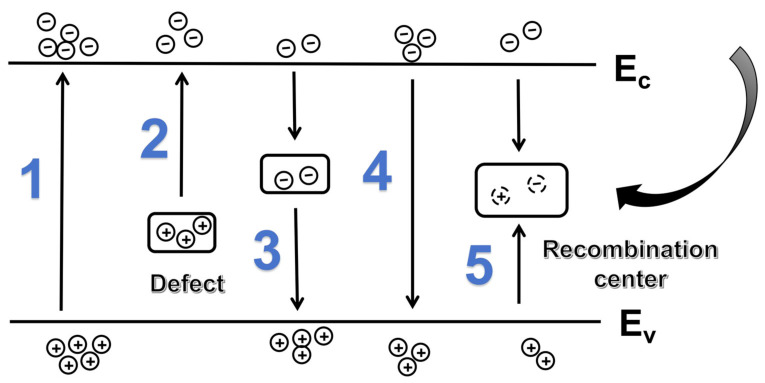
Schematic diagram of the carrier transport mechanism in the EBD sample.

The photoresponse mechanism of semiconductor materials involves complex photogenerated carrier dynamics, including the generation, trapping, and recombination of electron–hole pairs [44]. The carrier transport model is shown in Figure 6. Under ultraviolet irradiation, carrier generation primarily occurs through direct transitions between the valence band and conduction band (Process 1) and defect-assisted indirect transitions (Process 2). Under 254 nm ultraviolet irradiation, carrier generation is dominated by direct interband transitions (Process 1), with only a small fraction occurring via indirect transitions involving defect states (Process 2). It is worth noting that some photogenerated carriers are captured by trap states within the film (Process 3). After illumination ceases, carriers undergo rapid recombination with valence-band holes either via the recombination center (Process 4) or through direct interband recombination (Process 5), forming the fast response component of the decay curve. Meanwhile, carriers trapped in deep energy level traps are slowly released and subsequently participate in the recombination process. Due to the typically extremely deep trap levels in wide bandgap semiconductors [45], the depth of these traps determines the duration of transient decay. This process exhibits significant long relaxation characteristics, corresponding to the slow response component of the decay curve [46].

Significant slow response components were observed in the EB samples studied here, attributed to the substantial influence of numerous deep-level trap states in the material on carrier recombination dynamics [46]. In addition, we subjected ε-Ga_2_O_3_ to high-temperature annealing to obtain a gallium oxide thin film containing both ε and β phases, which we named THEB. The optical response curve measured under 254 nm laser (Supporting Information Figure S3) indicates that, unlike sample EB, sample THEB exhibits no memristive characteristics. The output curves at 254 nm, 365 nm, and under dark conditions indicate that the conductivity of sample THEB is significantly lower than that of sample EB. Particularly under no illumination and 365 nm laser irradiation, sample THEB exhibits near-insulating properties. This indicates that the annealed ε/β mixed-phase gallium oxide films exhibit significantly fewer defect states compared to those obtained through direct growth, and thus lack the abundant deep-level trap states necessary to manifest pronounced memristive characteristics.

## 5. Conclusions

This study demonstrates a photoresistive memristor based on the wide-bandgap material gallium oxide, integrating functions such as optical synapses and data storage. CVD technology has been successfully applied to deposit epsilon-Ga_2_O_3_ and beta-Ga_2_O_3_ films on sapphire (0001) substrates. This study further utilized CVD technology to fabricate beta/epsilon mixed-phase Ga_2_O_3_ films. By comparing three distinct gallium oxide films, it was discovered that the film exhibiting both beta and epsilon crystalline phases demonstrates significant absorption under deep ultraviolet light at 254 nm wavelength. It also exhibits solar-blind sensitivity characteristics. Compared to pure epsilon-phase and beta-phase gallium oxide, it exhibits more pronounced memristive properties. Further testing demonstrates that the MSM structure fabricated from this film exhibits outstanding optoelectronic synaptic functionality as a synapse device and memory unit, including STP, LTP, and “learning-forgetting-relearning” processes. Benefiting from the synergistic properties of two gallium oxide crystal phases, the device exhibits outstanding non-volatility. Notably, its resistive state retention time exceeds 104 s, coupled with long-term stability on an annual scale (>1 year). This achievement lays a crucial foundation for developing advanced neuromorphic systems that simulate long-term memory and sustained learning. Looking ahead, the introduction of paired spike-timing-dependent plasticity (STDP)-like learning rules in such devices, thereby further simulating the temporal plasticity observed in biological synapses. Based on the photonic–electrical synergistic properties of gallium oxide, the all-optical artificial synapse demonstrated significant potential in the field of neuromorphic computing.

## Data Availability

The original contributions presented in this study are included in the article/Supplementary Material. Further inquiries can be directed to the corresponding author.

## Supplementary Materials

The following supporting information can be downloaded at: https://www.mdpi.com/article/10.3390/ma19040711/s1, Figure S1: Optical characterization of samples E, EB, and B. (a) Three-dimensional AFM images of samples E, EB, and B; (b) SEM images of samples E, EB, and B; (c) Optical images of devices fabricated from samples E, EB, and B. Figure S2: Output curves and photoresponse curves of the EB sample after one year of storage. (a) Output curves under 254 nm, 365 nm, and no light illumination. (b) Single photoresponse curve to 254 nm UV light at 10 V. (c) Multiple photoresponse curves to 254 nm UV light at 10 V; Figure S3: Output curves and photoresponse curves of THEB samples. (a) Linear plots of output curves under 254 nm, 365 nm, and no light illumination. (b) Logarithmic plots of output curves under 254 nm, 365 nm, and no light illumination. (c) Multiple photoresponse curves to 254 nm UV light at 10 V.
