# Supplementary material for: All-Optical Artificial Synapse Based on ε-Ga2O3 and β-Ga2O3 Mixed-Phase Thin Films
Source: Materials (Basel). 2026 Feb 12;19(4):711. doi: 10.3390/ma19040711 (PMC12942465; doi:10.3390/ma19040711)
Supplement: Supplementary file 1 [file materials-19-00711-s001.zip › Supplementary material.pdf]

Supplementary material

All-Optical Artificial Synapse Based on ε-Ga_2O_3 and β-Ga_2O_3 Mixed-Phase Thin Films, by Jiale Niu et. al.

Section S1: Optical Characterization of Sample E, Sample EB, and Sample B

This study employed chemical vapor deposition to prepare the samples. The electrodes were formed directly on the sample surfaces by manually dispensing silver paste, making precise control of the silver paste thickness challenging. The specific dimensions of the sample are shown in Figure S1(c). Figure S1(a) and (b) show the AFM and SEM images of the three samples, respectively. Analysis of Figure S1(b) yields the root-mean-square roughness (Rq) values of 2.984 nm, 0.109 nm, and 0.057 nm for samples E, EB, and B, respectively. Figure S1(c) shows the film thicknesses of Sample E, Sample EB, and Sample B to be 823 nm, 903 nm, and 992 nm, respectively.

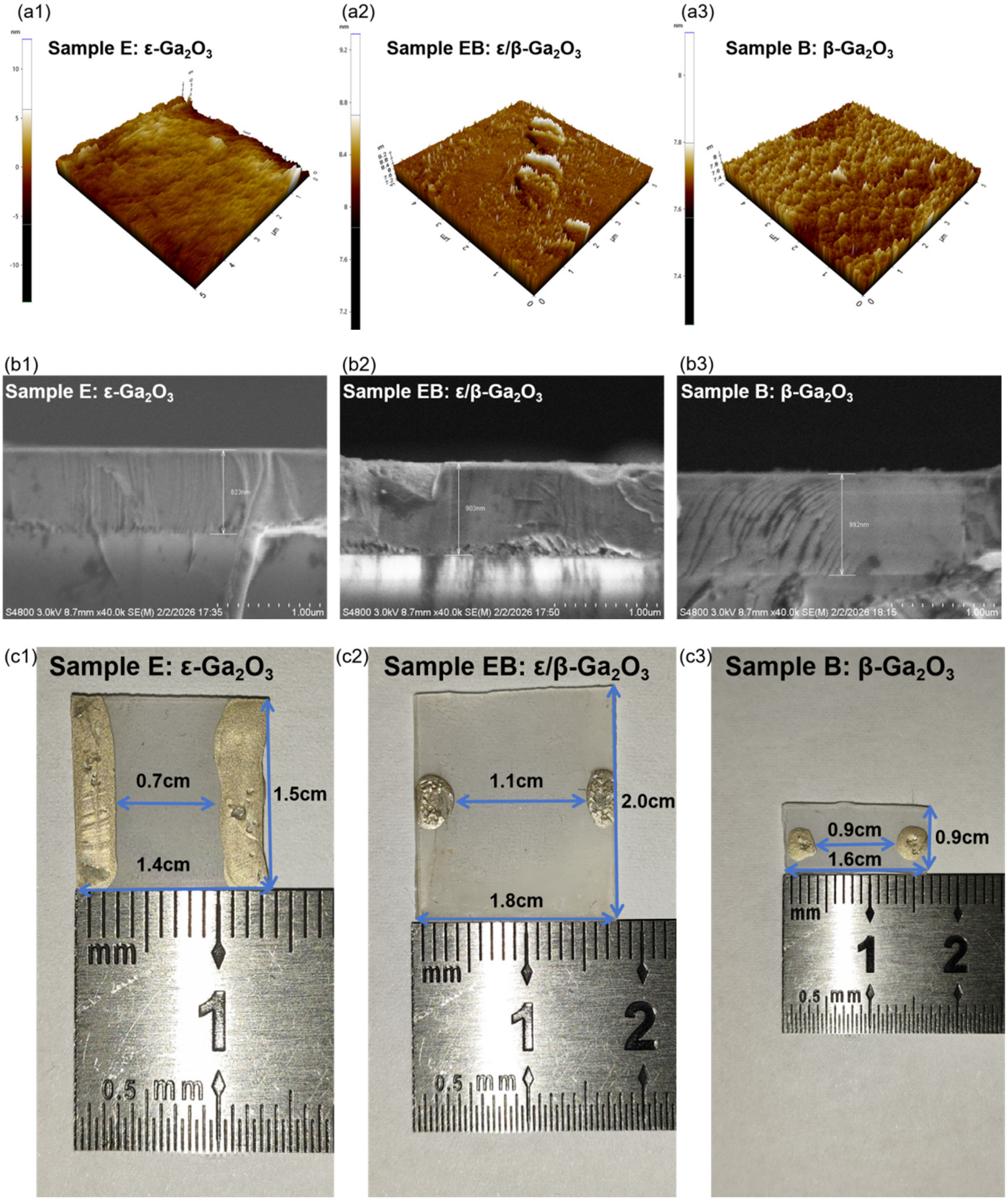

Figure S1. Optical characterization of samples E, EB, and B. (a) Three-dimensional AFM images of samples E, EB, and B; (b) SEM images of samples E, EB, and B; (c) Optical images of devices fabricated from samples E, EB, and B.

Section S2: Air Stability Testing of Mixed-Phase Gallium Oxide Thin Films Containing Both ε Phase and β Phase

We conducted performance retesting on sample EB, which had been stored for over a year. Figure S2(a) shows the output curves of the sample under 254 nm, 365 nm, and no illumination conditions. Compared with Figure 2(b), it can be observed that after long-term storage, the overall photocurrent of the sample shows a slight decreasing trend. Further comparison between Figures S2(b), (c) and Figures 3(e), (b) reveals that although the magnitude of photocurrent variation has decreased, the difference in photocurrent under different illumination conditions remains extremely limited. This indicates that the optical response characteristics are largely preserved. Overall, the samples exhibited no significant performance degradation after long-term storage. Furthermore, the slight fluctuations in photocurrent may also be partially attributed to minor variations in the contact position between the measurement device and the electrodes during testing.

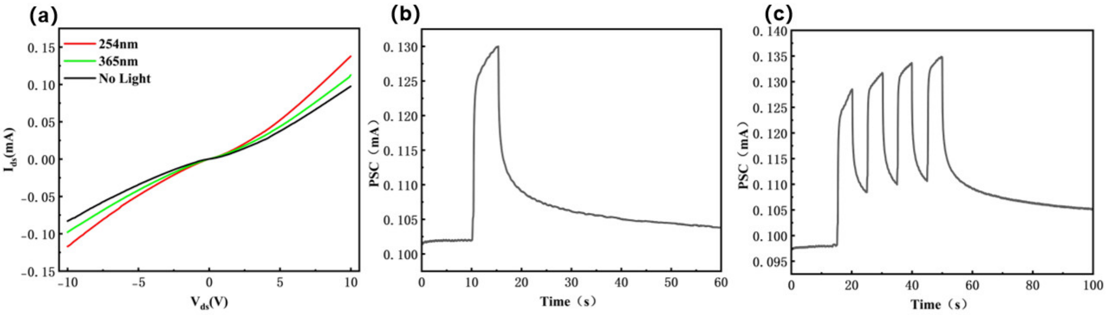

Figure S2. Output curves and photoresponse curves of the EB sample after one year of storage. (a) Output curves under 254 nm, 365 nm, and no light illumination. (b) Single photoresponse curve to 254 nm UV light at 10 V. (c) Multiple photoresponse curves to 254 nm UV light at 10 V.

Section S3: Comparison between annealed gallium oxide mixed-phase films containing both ε and β phases and directly grown gallium oxide mixed-phase films

β-phase gallium oxide can be obtained from the ε phase through annealing. Based on this method, we annealed ε-Ga_2O_3 films to prepare mixed-phase films (designated as sample THEB) and investigated the differences between the annealed films and mixed-phase films obtained by direct growth. Figures S3(a) and S3(b) show the linear and logarithmic coordinate output curves of the sample THEB under 254 nm, 365 nm, and no illumination conditions, respectively.Comparing with Figures 2(e) and (b), it can be observed that the conductivity of the annealed sample THEB is significantly lower than that of sample EB. Particularly under no illumination and 365 nm laser irradiation, sample THEB exhibits near-insulating properties, indicating a substantial reduction in defect density compared to sample EB. This is primarily due to the significant disappearance of defects (such as oxygen vacancies) that were originally uniformly distributed within the ε phase during the high-temperature annealing process. Newly generated defects primarily cluster at a small number of relatively coarse phase boundaries and grain boundaries, and may introduce macroscopic defects—microcracks. The formation mechanism is as follows: High-temperature annealing induces a phase transformation from the meta-stable ε phase to the stable β phase. This phase transformation is accompanied by lattice volume expansion. However, since the film is constrained by the substrate and cannot undergo lateral expansion, this leads to the generation of enormous internal stresses. When the stress exceeds the material's strength limit, cracking occurs to release energy. The micro-cracks introduced by annealing disrupt the macroscopic continuity of the material, making it difficult to sustain the switching behavior of direct-current memristors that relies on long-range continuous conductive pathways. This also explains the phenomenon observed in Figure S3(c), where the photocurrent exhibited almost no memristive

behavior after multiple stimulations with a 10 V bias and a 254 nm laser.

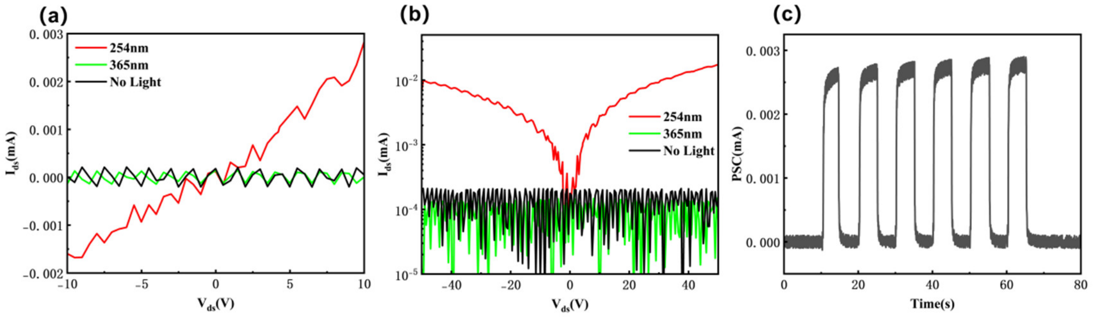

Figure S3. Output curves and photoresponse curves of THEB samples. (a) Linear plots of output curves under 254 nm, 365 nm, and no light illumination. (b) Logarithmic plots of output curves under 254 nm, 365 nm, and no light illumination. (c) Multiple photoresponse curves to 254 nm UV light at 10 V.
